# The MET Family of Receptor Tyrosine Kinases Promotes a Shift to Pro-Tumor Metabolism

**DOI:** 10.3390/genes15070953

**Published:** 2024-07-20

**Authors:** James C. Davis, Susan E. Waltz

**Affiliations:** 1 Department of Cancer Biology, College of Medicine, University of Cincinnati, Cincinnati, OH 45267, USA; 2 Research Service, Cincinnati Veterans Affairs Medical Center, Cincinnati, OH 45220, USA

**Keywords:** RTK, RON, c-MET, MET, MST1R, cancer, metabolism

## Abstract

The development and growth of cancer is fundamentally dependent on pro-tumor changes in metabolism. Cancer cells generally shift away from oxidative phosphorylation as the primary source of energy and rely more heavily on glycolysis. Receptor tyrosine kinases (RTKs) are a type of receptor that is implicated in this shift to pro-tumor metabolism. RTKs are important drivers of cancer growth and metastasis. One such family of RTKs is the MET family, which consists of MET and RON (MST1R). The overexpression of either MET or RON has been associated with worse cancer patient prognosis in a variety of tumor types. Both MET and RON signaling promote increased glycolysis by upregulating the expression of key glycolytic enzymes via increased MYC transcription factor activity. Additionally, both MET and RON signaling promote increased cholesterol biosynthesis downstream of glycolysis by upregulating the expression of SREBP2-induced cholesterol biosynthesis enzymes via CTTNB1. These changes in metabolism, driven by RTK activity, provide potential targets in limiting tumor growth and metastasis via pharmacological inhibition or modifications in diet. This review summarizes pro-tumor changes in metabolism driven by the MET family of RTKs. In doing so, we will offer our unique perspective on metabolic pathways that drive worse patient prognosis and provide suggestions for future study.

## 1. Introduction

Cancer is the second most common cause of death in the U.S. In 2023, it was estimated that more than 1.9 million new cancer diagnoses and around 600,000 deaths from cancer would occur [1]. To limit the number of deaths, it is important to identify the molecular drivers of cancer.

A key mechanism that drives the growth of cancer is a shift to a pro-tumor form of metabolism [2]. Non-cancerous cells rely primarily on oxidative phosphorylation for ATP production, while cancer cells rely much more heavily on glycolysis for ATP production, a phenomenon known as the Warburg effect [2]. In addition to increased glucose flux, in cancerous cells, pyruvate is reduced into lactate at a higher rate than in noncancerous cells [3]. Transport of lactate out of the tumor cell into the tumor microenvironment has multiple functions. Lactate can act as an important energy source and promote the polarization of macrophages into a pro-tumor state [4]. Thus, the shift to a pro-tumor form of metabolism produces more energy and helps maintain a preferential microenvironment for increased tumor growth, metastasis, and treatment resistance [5]. Therefore, targeting pro-tumor metabolism is an important opportunity to treat cancer.

Receptor tyrosine kinases [RTKs] are a type of protein that helps facilitate the shift to a pro-tumor metabolism. RTKs are signaling molecules that exist in the plasma membrane as monomers. Upon overexpression of the receptor or stimulation with the receptor’s ligand, receptor monomers dimerize either as homodimers or heterodimers and induce downstream signaling via direct and indirect phosphorylation of target proteins [6]. There is no known family of RTKs that do not dimerize. There are around 20 families of RTKs and in this review we will focus on the MET family of RTKs. In our discussion, we will compare the MET family’s role in pro-tumor metabolism with one of the most well-characterized RTKs, EGFR.

The MET family of RTKs consists of two members, MET and RON. These two receptors share 25% homology in their extracellular regions and 68% homology in their intracellular regions [7]. The respective ligands for MET and RON, hepatocyte growth factor [HGF] and hepatocyte growth factor-like protein [HGFL] share 43% homology [8,9,10]. Overexpression of either MET or RON is associated with worse patient prognosis in a variety of tumor types [11,12,13]. In addition to overexpression, MET can also influence tumor growth via over-activation, MET gene amplification, fusion of MET with another gene, or mutations that activate MET [14,15]. Recent research in cancer has identified a link between MET and RON and the development of pro-tumor metabolism [16,17,18]. This review summarizes the current knowledge of how the MET family induces pro-tumor metabolism and provides potential targets to limit MET family-driven cancer growth and metastasis.

## 2. MET Family Signaling in Pro-Tumor Glycolysis

Glycolysis is a series of enzyme-driven chemical reactions that convert glucose into pyruvate [19]. Along the way, NAD^+^ is reduced to NADH and two molecules of ATP are produced. In normal cells, pyruvate is preferentially pushed into the citric acid cycle in the form of acetyl-CoA, in a series of reactions that produces more NADH [20]. This NADH is then used in the mitochondria to produce ATP via the electron transport chain. The electron transport chain is dependent on oxygen as the final electron acceptor, so in situations where oxygen levels are low, the electron transport chain cannot function as efficiently. In cancer cells, pyruvate is reduced preferentially into lactate in a reaction that produces NAD^+^ from NADH, regenerating the NAD^+^ utilized in glycolysis. This preference towards lactate production from pyruvate helps drive tumor growth, as cancer cells are no longer as reliant on oxygen for their energetic needs. Thus, cancer cells thrive in low oxygen environments, which allows tumors to bigger without requiring vascularization. Even in high oxygen environments, cancer cells preferentially utilize glycolysis for their energetic needs, an aberrant metabolic state known as aerobic glycolysis. Various inhibitors have been developed to target glycolysis, such as 2-DG and metformin, although the effectiveness of these molecules has yet to be established [21].

**Figure 1:**
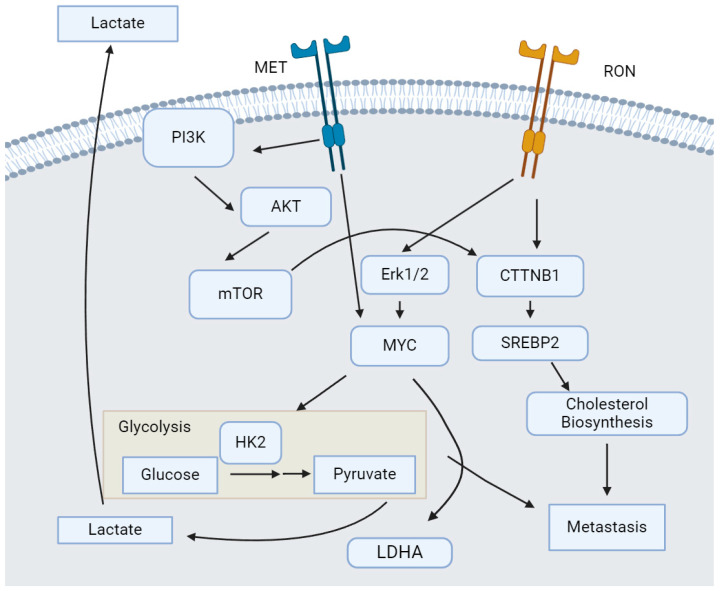
**MET and RON promote glycolysis and cholesterol biosynthesis leading to increased metastasis.** RON and MET activate MYC transcription factor activity which increases the expression and activity of glycolytic enzymes. RON and MET both signal through CTTNB1 to induce SREBP2-mediated cholesterol biosynthesis.

Both MET and RON have been implicated in the shift towards aerobic glycolysis in tumor cells. The activation of MET by HGF is associated with increased glucose utilization and increased glycolytic enzyme expression [22,23,24]. In pancreatic ductal adenocarcinoma [PDAC], pancreatic stellate cells in the tumor microenvironment secrete HGF which activates MET and induces HK2 expression in PDAC cells in a paracrine manner [17]. This induction of HK2 expression is ameliorated with the addition of PHA-665752, a MET inhibitor. The link between HK2 expression and MET activation appears to be dependent on the cell line studied, as another group studying MET in head and neck cancer found that HGF treatment only induced HK2 and LDHA expression in one out of the three cell lines tested [22]. They found that this cell line, Detroit 562, had much higher levels of MET expression than the other two cell lines. Thus, the overexpression of MET is an important factor in the induction of HGF-mediated glycolytic enzyme expression. Interestingly, RON expression has also been associated with increased glycolysis by upregulation of HK2 and LDHA, implying a conserved function in regulating glycolysis between the two members of the MET family [16]. In addition, short-form RON, a transcript variant of RON, has been shown to activate glycolysis better than the full-length RON transcript [25]. Both MET and RON activate glycolysis by phosphorylating ERK1/2 at Tyr204 stabilizing MYC protein and thus increasing the expression of MYC transcriptional targets (Figure 1) [16,23,26]. 

RONΔ160 is an isoform of RON that is linked to decreased ERK1/2 phosphorylation, so it would be expected that RONΔ160 would promote glycolysis much less than full-length RON [27]. However, short-form RON has also been linked to decreased ERK phosphorylation, implying that short-form RON may promote glycolysis via a different mechanism than full-length RON [28]. This means that although there is decreased ERK1/2 phosphorylation from RONΔ160 expression, the possibility remains that RONΔ160 may promote glycolysis better than full-length RON. An isoform of MET, Δ13MET, also shows decreased ERK1/2 phosphorylation compared to full-length MET, although more research is required to understand how this isoform affects glycolysis [29].

MET activity increases both glycolytic activity and oxidative phosphorylation [24], while cells with RON and without RON have the same decrease in oxygen consumption rate upon addition of oligomycin, an inhibitor of the electron transport chain [16]. Thus, MET may induce a more general increase in energy production via glycolysis and oxidative phosphorylation, while RON only increases glycolysis. We have shown that RON-expressing cells secrete more lactate than isogenic cells with low RON levels [30]. This is a potential mechanism by which RON-expressing tumors promote a more favorable tumor microenvironment, as extracellular lactate can promote anti-inflammatory macrophage polarization [4]. Although there is no evidence in the literature supporting MET’s role in lactate secretion, like RON, MET increases LDHA levels and may contribute to increased lactate levels in the tumor as more lactate is produced upon MET activation [22]. EGFR, like both MET and RON, promotes aerobic glycolysis and lactate production [31]. In addition, unlike RON or MET, inhibition of EGFR reactivates oxidative phosphorylation [32]. These differences highlight the wide variety of effects that different RTKs can have on glycolysis.

While knowledge of the role that RON has in regulating glycolysis is limited, there is much more evidence of MET’s importance in glycolysis. ENO1, a glycolytic enzyme, has been shown to phosphorylate MET, promote WNT activation, and inactivate the β-catenin destruction complex [33]. This then helps drive epithelial-to-mesenchymal transition [EMT] in lung cancer, leading to increased metastasis. MET signaling also activates ERK1/2, which then promotes the nuclear translocation of the glycolytic enzyme PKM2 [26]. Nuclear PKM2 then phosphorylates histone H3 to promote the expression of CCND1 and MYC for increased growth and proliferation of retinoblastoma cells. In addition to the role of MET in increasing glycolytic enzyme expression, MET activity also inhibits PDH, limiting the flow of glycolytic products into the citric acid cycle and thus promoting the production of lactate via LDHA [34]. High levels of glucose can also induce MET signaling and promote an aggressive cancer phenotype with increased EMT [35]. This showcases the role that diet may play in the aggressiveness of tumors, as foods inducing high levels of blood glucose may promote MET activation and worsen patient prognosis. 

## 3. MET Family Signaling in Cholesterol Metabolism

Cholesterol biosynthesis is a multi-step enzymatic process in which acetyl-CoA is converted into cholesterol [36]. While cholesterol can still be taken in from the tumor microenvironment, tumor cell intrinsic cholesterol biosynthesis promotes metastasis and recurrence via CCDC25 [37]. Metabolites of cholesterol, such as 25-hydroxycholesterol and 27-hydroxycholesterol, can activate ERβ, leading to increased metastasis [38,39]. In addition, the reduction of cholesterol by CYP27A1 into bile salts lowers the levels of cholesterol in the cell and lowered bile salt production is associated with increased metastasis [40]. Increased cholesterol biosynthesis has been linked to the treatment-resistant, metastatic/recurrent cancer stem cell phenotype [41]. Although it is difficult to increase cholesterol-lowering processes such as bile salt production, the reliance of cholesterol biosynthesis on enzymes provides key areas for targeted inhibition to lower cholesterol levels. HMGCR is the rate-limiting enzyme in the mevalonate cholesterol biosynthesis pathway that can be targeted with the FDA-approved class of inhibitors known as statins [42]. Statin treatment has been shown to reduce metastasis, recurrence, and increase survival in multiple types of cancer [43,44,45,46]. Although the process of cholesterol biosynthesis has been studied in its role in cancer metastasis, much less is known about the drivers of cholesterol biosynthesis in cancer. 

MET and RON both upregulate cholesterol biosynthesis via signaling through CTTNB1 to induce SREBP2 transcription factor activity (Figure 1) [16,18,47]. MET, in particular, signals through the PI3K/AKT/mTOR axis to induce CTTNB1 phosphorylation driving SREBP2 activity [18,41,47], while RON appears to directly phosphorylate CTTNB1 at Tyr654 and Tyr670 [48]. SREBP2 then drives transcription of cholesterol biosynthesis genes such as SQLE, LSS, and HMGCR, leading to increased cholesterol biosynthesis [49]. The increased levels of cholesterol biosynthesis induced by RON signaling promote metastasis and recurrence [16]. Using statins to treat mice with RON-expressing tumors limits both metastasis and recurrence [16]. In addition, HGF-induced MET signaling inhibited CYP27A1 and decreased the conversion of cholesterol into bile salts, showcasing the potential benefits on cholesterol levels of directly targeting members of the MET family rather than only targeting cholesterol biosynthesis with statins [50]. It is not yet clear if RON has any role in the catabolism of cholesterol, but the effects of RON on cholesterol biosynthesis make it another important target in limiting metastasis and recurrence.

While MET and RON promote cholesterol biosynthesis, very little is known about the role that EGFR may play in promoting cholesterol biosynthesis. Constitutively active EGFR activates Src/Erk/YTHDF2 and inhibits LXRα [51]. Inhibition of LXRα promotes cholesterol efflux and inhibits LDL uptake. Thus, active EGFR would be expected to lower cellular cholesterol levels. However, it was found that overexpression of YTHDF2 resulted in increased cellular cholesterol levels. This implies that although cholesterol intake was suppressed and efflux was increased, cholesterol levels may be increased from enhanced cholesterol biosynthesis. In addition, while little is known about how cholesterol influences RON and MET expression, evidence in the literature indicates that cholesterol influences EGFR protein levels. Cholesterol depletion releases EGFR from lipid rafts, which both relieves functional inhibition of EGFR and promotes EGF-independent EGFR signaling [52,53]. Other evidence in the literature suggests that cholesterol suppresses the degradation of EGFR and promotes EMT in prostate cancer [54]. In addition, cholesterol promotes EGFR inhibitor resistance via the EGFR/ERRα axis [55]. Thus, the effect of cholesterol on MET RTK family activity and expression presents a novel route for further research.

## 4. The Role of MET Family Proteins in Other Metabolic Pathways

### 4.1. Glutamine Metabolism

In cancer, the breakdown of glutamine into glutamate by GLS provides an ammonium group for biosynthetic reactions [56]. Glutamate is also used alongside cysteine and glycine to produce glutathione, the major scavenger of ROS. Glutathione (GSH) is essential in cells to limit ROS to non-lethal levels and excess GSH promotes metastasis and growth [57]. GSH sequesters ROS via oxidation of two GSH molecules into GSSG. MET promotes GLS expression in liver cancer leading to decreased GSSG:GSH ratio, implying that there may be an excess of GSH when MET is activated [34]. This excess in GSH would promote metastasis and growth, leading to worse outcomes when MET is activated. In contrast, there is very little known about how RON influences glutamine metabolism, but the evidence that exists suggests that the loss of RON leads to decreased glutamine, increased GSH levels, and little effect on glutamate levels [30]. Thus, while MET increases GSH levels, RON decreases GSH levels which one would expect to decrease the metastatic capacity of cells that express RON. Cells that express RON have higher metastatic capacity which means that other factors downstream of RON expression have a greater role in metastasis than GSH. EGFR inhibitor-resistant cell lines have lower levels of GSH compared to EGFR inhibitor-sensitive cell lines [58]. When supplied with exogenous GSH, these resistant cell lines gain sensitivity to EGFR inhibitors. This change in treatment resistance highlights the potential benefit of nutritional supplementation on patient outcomes, although more research is required in in vivo models to grasp the full extent of possibilities.

### 4.2. Asparagine Metabolism

Asparagine is an amino acid that is synthesized starting with the incorporation of glutamate into the citric acid cycle [59]. Transamination of oxaloacetate results in aspartate, which is then modified by ASNS into asparagine [60]. Although the exact role of asparagine in cancer is not well understood, most of the evidence links ASNS expression to proliferation and metastasis. In fact, ASNS expression promotes the expression of MET in non-small cell lung cancer cells [61]. More research needs to be carried out in discerning how ASNS promotes MET expression, but the potential of inhibiting ASNS to limit MET expression is a tantalizing avenue of treatment. Loss of RON leads to increased aspartate levels, which implies that either aspartate synthesis is increased in cells without RON or that ASNS is not as active, leading to accumulation of aspartate [30]. Asparagine levels have been targeted with positive outcomes in patients with acute lymphoblastic leukemia via the use of L-asparaginase [62]. This showcases the use of an enzyme to break down a pro-tumor metabolite, a method of treatment that shows great promise.

### 4.3. Pentose Phosphate Pathway

The pentose phosphate pathway [PPP] shifts glycolytic products away from glycolysis and into ribonucleotide and NADPH synthesis, two molecules required for rapidly growing cancer cells [63]. The third enzyme involved in the PPP is 6GPD, an enzyme that catalyzes the production of NADPH and ribulose-5-phosphate from 6-phosphogluconate and NADP [64]. The expression of 6GPD promotes MET phosphorylation and cell growth and migration [65]. This association of 6GPD and MET highlights the interconnectivity of metabolism and receptor activity. 

### 4.4. Oxidative Phosphorylation

Although most cancer cells rely primarily on glycolysis for energy production, there is evidence that in certain tumor types oxidative phosphorylation is a key pathway for energy production and starting point for various anabolic reactions [66]. Oxidative phosphorylation has been linked to increased metastatic features [67,68]. In line with this phenotype, MET increases the activity of complex 1 in the electron transport chain via the MET/ERK/STAT3 axis [69]. Mitochondrial fusion and fission both appear to be required for effective cancer metastasis [70]. MET directly phosphorylates FIS1, which drives increased mitochondrial fission and metastasis [71] EGFR, however, promotes mitochondrial fusion [72]. This difference highlights how both the forward and reverse of a process that influences metabolism can be pro-tumor.

### 4.5. Fatty Acid Metabolism

**Figure 2:**
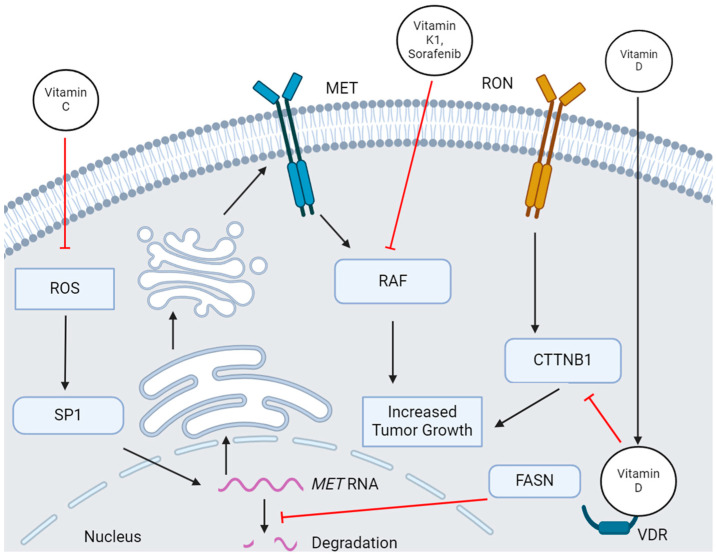
**The effect of Vitamins on MET activity.** Vitamin K1 and sorafenib inhibit MET-mediated RAF activity, decreasing tumor growth. Vitamin C lowers ROS and limits SP transcription factor activity leading to less production of MET RNA while FASN inhibits the degradation of MET RNA. Vitamin D inhibits CTTNB1 via VDR leading to an inhibition of tumor growth.

The synthesis of fatty acids driven by FASN has been implicated in worse prognosis in various types of cancer [73,74]. In addition, various papers link FASN expression with cholesterol levels [73,75]. Other research has shown that MET expression is correlated with FASN expression, and that inhibition of FASN is linked to post-transcriptional downregulation of MET mRNA levels (Figure 2) [76,77]. Although a direct causal link for a FASN/MET/cholesterol axis has not been established, the role of MET in regulating cholesterol biosynthesis implies that the regulation of cholesterol by FASN may be a downstream effect of the FASN/MET axis. In contrast, while little is known about the effect of FASN on EGFR, EGFR in the plasma membrane can drive FASN-and ACLY-mediated palmitate synthesis which promotes cellular survival [72]. Thus, FASN inhibition provides an avenue by which pro-tumor effects of both MET and EGFR can be targeted.

## 5. The Therapeutic Role of Vitamins in MET Family-Expressing Cancers

The role of diet in cancer prognosis is an under-studied area of interest. It can be hard for patients to maintain a specific diet due to side effects of cancer treatment. This can make it difficult for therapeutic interventions to be introduced through changes in diet. Nevertheless, diet is quickly becoming an important target of cancer research [78]. Vitamins are a key part of diet that act as co-factors in many reactions and can have their own dedicated receptors that can affect cell growth and viability.

The activity of MET and RON are both affected by vitamins. Vitamin D3-induced activation of Vdr in the MMTV-RON-driven model of breast cancer leads to increased time to tumor initiation and decreased metastasis via inhibition of Cttnb1 downstream of RON signaling (Figure 2) [79]. Loss of Vdr in this model decreases active Cttnb1 levels and decreases time to tumor initiation. Inactivation of Cttnb1 by Vitamin D3 supplementation provides a potential alternative to statin treatment in limiting cholesterol biosynthesis. In contrast, activation of MET via HGF with the addition of Vitamin D inhibits growth in androgen-unresponsive prostate cancer cell lines [80]. The mechanism of action by which this growth inhibition occurs has yet to be elucidated. In some models, Vitamin D supplementation promotes HGF secretion leading to increased senescence and decreased tumor growth [81]. Although more research is required to understand the mechanism, the secretion of HGF induced by Vitamin D implies that HGF is a target gene of VDR. This points to the potential senescent effects of MET. Other researchers have observed no change in growth in mutant CTTNB1 MET-overexpressing hepatocellular carcinoma with the addition of Vitamin D [82]. This implies that the mechanism by which Vitamin D inhibits growth in both RON-expressing and MET-expressing cancers is via inhibition of CTTNB1. 

Retinoic acid, a metabolite of Vitamin A, modulates both HGF, HGFL, and EGFR expression. Retinoic acid activates RAR to induce downstream signaling. In the hepatocellular carcinoma cell line, HepG2, treatment with all-trans-retinoic acid represses the expression of HGFL [83]. The researchers found that the HNF-4 binding sequence in the HGFL promoter was crucial for this repression. Even though the levels of HNF-4 bound to the HGFL promoter was not changed, they found that overexpression of CBP, a transcriptional co-activator, rescued the expression of HGFL when the cells were treated with all-trans retinoic acid. The expression of EGFR in the human breast cancer cell line SkBr3 was found to be reduced upon treatment with all-trans-retinoic acid or 9-cis retinoic acid [84]. Other research into retinoic acid’s effect on HGF expression showed similar results [85]. In fact, in an astrocytoma cell line, U87, treatment with other agonists of the retinoic acid receptor resulted in a decrease in HGF expression and secretion. This implies that the repression of HGF, HGFL, and EGFR may be RAR-mediated rather than from the effects of retinoic acid as a metabolic cofactor. 

While the extent of our knowledge on the effects of vitamins on RON signaling is limited to Vitamin D, more research has been published on the effects of vitamins on MET signaling. Vitamin C lowers reactive oxygen species [ROS] in tumor cells, decreasing the activity of SP transcription factors and thus the SP-dependent expression of MET and EGFR (Figure 2) [86]. Since SP1 is an essential transcription factor for RON expression, it would be expected that Vitamin C treatment would also decrease the expression of RON via the same mechanism [87]. Vitamin K3 induces phosphorylation of both MET and EGFR and strongly induces ERK phosphorylation leading to decreased cell growth [88]. This is another case in which RTKs act in a manner that promotes senescence, as functional readouts of MET and EGFR signaling such as GRB2/MET association and GRB2/EGFR association are increased upon Vitamin K3 treatment along with decreased growth. The effect of Vitamin K on MET phosphorylation in combination with sorafenib, a kinase inhibitor, is much less clear. In hepatocellular carcinoma, Vitamin K and sorafenib treatment synergistically decreased MET phosphorylation, leading to decreased EMT, proliferation, and migration [89]. Other researchers found that Vitamin K and sorafenib treatment in hepatocellular carcinoma promotes inhibitory MET Y1349 phosphorylation, leading to increased PI3K/AKT signaling and inhibition of RAF (Figure 2) [90]. Although some researchers found MET phosphorylation to be decreased in response to Vitamin K/sorafenib treatment, it is important to note that the phospho-residue measured was not disclosed [91]. Thus, it is possible that Vitamin K/sorafenib treatment decreases activating MET phosphorylation while increasing inhibitory MET phosphorylation. While it has not been tested in regard to MET or RON, Vitamin E has been shown to decrease EGFR levels, indicating the possibility that other untested vitamins may have an effect on MET and RON signaling [92]. 

## 6. Summary and Future Directions

Current research has identified key metabolic pathways and downstream players of RON and MET signaling that promote worse patient outcomes. RON and MET both induce glycolysis and cholesterol biosynthesis by MYC and CTTNB1. MET also induces oxidative phosphorylation via ERK, which presents a potential target for pharmacological inhibition. MET inhibits both PDH and bile salt production, which may play a role in increasing lactate and cholesterol levels. While RON decreases GSH levels, MET increases GSH levels. Regardless of how the levels of GSH change, however, both receptors promote worse prognosis, implying that other functions of RON may be more important for patient outcomes. In addition, Vitamin D treatment targets CTTNB1 downstream of both MET and RON. MET activity is influenced by Vitamin K, leading to decreased metastasis. Comparing the MET family of RTKs with EGFR, it appears that there are untested mechanisms by which metabolism can influence MET/RON RTK activity. In summary, MYC or ERK inhibition and Vitamin D supplementation provide avenues to target both MET and RON-induced glycolysis and cholesterol biosynthesis, which would lead to better patient outcomes. 

Another option is direct inhibition of either MET or RON by a small molecule inhibitor to target downstream pathways. While clinical trials with a RON inhibitor have not made it past Phase 2, there are various FDA-approved MET inhibitors already in use in the clinic [93,94,95]. Cabozantinib and crizotinib have been approved for use in the treatment of non-small cell lung cancer. Treatment with cabozantinib has been shown to decrease glycolytic gene expression, although its effects on other metabolic pathways downstream of MET have not been established [95]. Multiple studies mentioned in this review regarding EGFR indicate that nutritional supplementation can influence resistance to RTK inhibitors. Thus, more research is required to fully understand the benefits of MET or RON inhibition on pro-tumor metabolism.

Currently there are no FDA-approved ERK inhibitors, with Phase 2 trials of LY3214996 being terminated due to low efficacy [96,97]. Therefore, other avenues of treatment need to be explored for more immediate results. Statin treatment inhibits cholesterol biosynthesis and has been shown to be well-tolerated in cancer patients, leading to improved outcomes [98]. While cholesterol biosynthesis is easily targeted, there has been much less luck in the realm of glycolytic inhibition, although there are a few clinical trials in progress [99]. Using Vitamin D in cancer treatment has led to mixed results, with some studies indicating favorable prognosis and others indicating no change in prognosis [100]. Additionally, most of the research presented in this review is based on in vitro experiments and more research needs to be carried out to discern the viability of targeting metabolic pathways downstream of MET and RON signaling in cancer in vivo.

## Data Availability

Not applicable.
